# Physiochemical, Insecticidal, and Antidiabetic Activities of *Senna occidentalis* Linn Root

**DOI:** 10.1155/2020/8810744

**Published:** 2020-09-15

**Authors:** Tahani Osman Issa, Ahmed Ibrahim Mohamed Ahmed, Yahya Sulieman Mohamed, Sakina Yagi, Abdelrafie Mohamed Makhawi, Tarig Osman Khider

**Affiliations:** ^1^College of Applied and Industrial Sciences, University of Bahri, P. O. Box 1606, Khartoum, Sudan; ^2^Institute of Medicinal and Aromatic Plants National Centre for Research, Khartoum, Sudan; ^3^Department of Botany, Faculty of Science, University of Khartoum, P. O. Box 11115, Khartoum, Sudan

## Abstract

The present study aimed to investigate the physiochemical activities of *Senna occidentalis* (Linn) roots and phytochemicals as insecticidal (ethyl acetate and methanol) and antidiabetic (ethanolic extract) activities. Physicochemical properties were carried out by using Association of Official Analytical Chemist methods; thin layer chromatography was carried out according to the Stahl method. Larvicidal activity and LD_50_ were studied against the third instar of *Culex quinquefasciatus* mosquito larvae to detect and extract toxicity. The ethanolic extracts of the roots were orally tested at the dose 200 mg/kg for the hypoglycemic effect on induced hyperglycemia in normal rats, assessed in the ethanol extract, and were compared with diabetic control and standards glibenclamide 10 mg/kg. Physiochemical parameters showed high rate in the nitrogen-free extract (69.6%), curd fiber (14.5%), crude proteins (8.15%), ether extract (3.75%), and both ash and moisture (2%), and high concentrations values were found in potassium (43 mg/l) followed by phosphorous (28.5 mg/l), calcium (15 mg/l), sodium (3.65 mg/l), and magnesium (0.145 mg/l). In this part, phytochemical compounds showed high amount of alkaloids, triterpene, flavonoids, tannins, sugars, and few amount of anthraquinone glycosides. Thin-layer chromatography (TLC) studies different colored phytochemical constituted with different Rf values. All the spots are colored under UV light, but some are localized colorless after spaying. The ethyl acetate (EtAc) extract showed eight spots, and the methanol (MeOH) extract showed thirteen spots. The larvicidal activity showed that the ethyl acetate extract was safe against mosquito larvae with an LD_50_ value 1412.54 (*p* < 0.05), and the methanol extract had moderate larvicidal activity against mosquito larvae with an LD_50_ value 257.54 (*p* < 0.05), while the ethanolic extract of *Senna occidentalis* (L.) causes a favorable hypoglycemic activity when compared to control significant reduction by [53.15%, 32.87%, and 20.94%], respectively, as well as standard glibenclamide. Based on the various data of the physicochemical parameters, TLC spots, and phytochemical compounds of *Senna occidentalis* root, they could be used as references standards for manufacturing units of *Senna occidentalis* root larvicidal and antidiabetic drugs.

## 1. Introduction

Standardization of medicinal plants is an essential issue to be considered as a therapeutic drug for safety in health care. *Diabetes mellitus* is a disease responsible for many deaths around the world [1, 2]. *Senna occidentalis* (Linn), or *Cassia senna* or *Cassia occidentalis*, in Sudan, known as Soreib (Ar.), which belongs to the family “*Caesalpinioideae*,” is widely distributed in all of Sudan. *Senna occidentalis* roots are used traditionally in treating of diabetes and other ailments [3]. However, its adequate validation as therapeutic, specifically antidiabetic and hepatoprotective effects, has not been established [4]. *Senna occidentalis* is prescribed for use only as a laxative and bowel cleanser for the treatment the colon and in surgery [5]. The seeds of *Senna occidentalis* are toxic for the children [6, 7]. The various extracts of the whole plants were subjected to normal and alloxan-induced diabetic rats. Gidadoand his co-workers [4] and Verma et al. [8] studied the effect of *Senna occidentalis* leaves on diabetic rats. In traditional medicine, leaves were externally applied to wounds, sores, itch, cutaneous diseases, bone fracture, fever, ringworm, skin diseases, and throat infection and to cure sore eyes, hematuria, rheumatism, typhoid fever, and *tuberculosis* [9].

Insects, ticks, and mites are dangerous vectors of deadly pathogens and parasites, which may be considered as epidemics in the increasing world population of humans and animals [10–14]. Mosquitoes are the most important single group of insects in terms of public health importance, which transmit malaria, filariasis, dengue, and Japanese encephalitis [15, 16]. Reduction of the source of infection is an essential step in the control of mosquito-borne diseases [17], although synthetic organic insecticides have been produced and used to eliminate mosquitoes. The treatment of the disease vectors using synthetic insecticides has failed due to their efficiency in attaining physiological resistance and effect on nontarget organisms [18]. The phytochemical compound present in other parts of *Senna occidentalis* has been isolated and such compounds are reviewed as sennoside, anthraquinone glycosides, fatty oils, flavonoids, glycosides, galactomannan, polysaccharides, and tannins [8]. The insecticidal activity of the extracts of this plant on vector mosquitoes is not sufficiently reported [19].

The objective of the present work was to determine and investigate the efficiency of the crude extract of the root of *Senna occidentalis* for larvicidal activity against the *Culex quinquefasciatus* mosquito larvae, as well as antidiabetic, with reference to physiochemical activities.

## 2. Materials and Methods

### 2.1. Plant Materials Collection and Identification

*Senna occidentalis* roots were collected in September 2015 from Al-Debabat city, locality of Algoz, South Kordofan State, Sudan [20]. Samples were identified in the herbarium at the department of phytochemical and natural resource where herbarium no. k. 14/96 was deposited at the herbarium, National Center for Research Medicinal and Aromatic Plants Institute, Khartoum, Sudan.

### 2.2. Preparation of Plant Materials

The roots part were air-dried in shadow, powdered by using a locally made hammer mill, and then, the plant were weighted with electric balance and stored for further analysis.

### 2.3. Preparations of Crude Extracts

196 g of the dried plant was weighted and, then, extracted successively with n-hexane (for four hours), ethyl acetate, and methanol extracts (eighteen hours), and the single ethanolic extract was also extracted by using a shaker apparatus at room temperature. All extracts were air-dried and kept separately in universal bottles till used for subsequent experiments. Their physical appearance and percentage yields properties were tabulated in results. Percentage yields were calculated as follows:

(1) $$\begin{array}{c} \text{Yield}\left(\text{\%}\right)=\frac{\text{Weight of extract}}{\text{Weight of sample}}\times 100. \end{array}$$

### 2.4. Phytochemical Screening

The general phytochemical screenings were carried out according to the methods described by Harborne and Sofowora [21–23].

### 2.5. Thin Layer Chromatography

The results of the Thin-Layer Chromatography (TLC) were carried out by using the standard method described by Galib et al. and Stalh [24, 25].

### 2.6. Physiochemical Anaylsis

Physicochemical properties such as the proximate composition (moisture, crude protein, crude fiber, crude lipid, and nitrogen-free extract %) of the roots were investigated using the standard methods of the Association of Analytic Chemist (AOAC, 1990-2005) methods [26–29]. The mineral compositions of the root sodium and potassium contents were determined by using a flame photometer, while calcium and magnesium contents were determined by using an Atomic Absorption Spectrophotometer (1100 B Perkin-Elmer, Germany) and phosphorus was determined by the vandomolybdate standard colorimetric method [26].

### 2.7. Cytotoxicity Bioassays

#### 2.7.1. Preparation and Hatching of *Culex quinefasciatus*

*Culex quinefasciatus* egg rafts were collected from stagnant water in the Al kadarow area. The egg rafts were kept in an aluminum dish with 10.6 inches width and 1.6 inches depth containing distilled water till hatching, and the larvae were fed with fine powdered bread. Dead larvae were continuously removed to a void contamination of cultures with pathogens. The third instars larvae for experiments were used.

### 2.8. Experiment Toxicity Tests of *Culex quinefasciatus*

The larvicidal activity of the *Senna occidentalis* root was determined to investigate lethality bioassay against *Culex quinefasciatus mosquito* third instar larvae of *Senna occidentalis* roots. The cytotoxicity bioassay experiment was performed according to modification procedure described by Nguta et al. [30]. Thirty larvae were selected and transferred into three test tubes by means of a 23 cm disposable Pasteur pipette from an aluminum dish, each containing 5 ml of distilled water. The samples for the experiment were prepared by dissolving 20 mg of each different extracts in each 5 mL of dimethyle sulphoxide (DMSO). Appropriate amounts of this DMSO solution (5 *µ*L, 50 *µ*L, and 500 *µ*L) to give three different concentrations of ethyl acetate and methanol extracts (10 ppm, 100 ppm, and 1000 ppm ppm (or mg/L), respectively) were transferred into 100 ml test tubes (3 test tubes for each dose and 1 for control). Three replicates were prepared for each dose level and, then, dried further in vacuum for 1 h to remove DMSO completely. Control was prepared using only DMSO, and then, the thirty larvae were placed in each test tube. The surviving larvae were counted with the aid of a 3x magnifying glass, after 24 hours. The second count was performed after 72 hrs, and larvae were considered after dead when they fail to rise to the surface or settle motion less in the bottom of the test tube.

### 2.9. LD_50_ Determination

The lethal death fifty (LD_50_) of plant extracts resulting in 50% mortality of the larvae of *Culex quinefasciatus* LD_50_ and 95% confidence intervals were determined from the 72-hour counts, and the dose response data were transferred into LD_50_ analyzed by Finney [31]. Probit Analysis computer program was used to determine lethal death fifty (LD_50_) values. Potassium dichromate (K^₂^Cr^₂^O^₇^) was used as a positive control with an LD50 value 1.7 *μ*g/ml (toxic) and DEMSO (1000 *μ*g/ml) as a negative control in the bioassay experiments (LD50 726129 × 10^11^*μ*g/ml) according to the modification by Meyer et al.. LD_50_ values below 250 *μ*g/ml were considered as highly toxic, 250–499 *μ*g/ml as moderately toxic, and 500–1000 *μ*g/ml as lowly toxic. Values above 1000 *μ*g/ml were regarded as safe or nontoxic according to McLaughlin and co-workers [32].

### 2.10. Diabetics Assay

#### 2.10.1. Study of Hypoglycemic Effects on Induced Hyperglycemia in Rats as Experimental Animals

**Figure 1:**
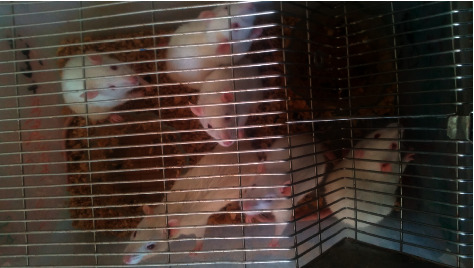
Experimental male albino rats.

Twenty-four healthy adult male albino strain rats weighing 150–250 g aged between 6–12 months (Figure 1) were purchased from the department of animal house laboratory, Faculty of Veterinary Medicine, Khartoum, Sudan. The rats were kept in a well-aerated laboratory and were divided into four groups with six rats each in cages. The animals were fed freely on standard animal feeds with balanced diet and drinking water *ad libitum* during the experimentation period. The animals were exposed to a 12 h light-dark cycle.

### 2.11. Experimental Design of the Glucose Loading Induction Model

Experimental rats were fasted from food for eighteen hours prior to experimentation and had water *ad libitum*. The first group (I) was administered with 10 ml/kg body weight of distilled water and considered as the normal control group (I). The second group (II) was administered with 2 g/kg body weight of glucose loading and considered as positive control. The third group (III) was administered with 10 mg/kg body weight of the hypoglycemic drug-glibenclamide and considered as the standard group. The last groups (IV) were treated with 200 mg/kg body weight the ethanolic extract of *Senna occidentalis* roots. All four groups of rats were, then, loaded with 50% glucose solution at a dose of 2 g/kg. The blood glucose level of all groups was monitored after 1, 2, and 4 hours after the glucose load according to Konuklugil et al. [33].

### 2.12. Determination of the Blood Glucose Level

The fasting blood glucose levels were first determined at “0” minutes of initial fasting blood samples which were collecting from the overnight fasted rats, by drawing out about 1 ml of the blood samples from eye side (retro-orbital plexus) cervical dislocation by using heparnized-capillary tubes under mild diethyl ether inhalation anaesthesia on a four-hour interval (starting with the fasting plasma glucose levels of the four groups of rats were determined (0 time), then 1, 2, and 4 hours) for a period of one day. A manual digital spectrophotometer was used to analyze blood glucose levels throughout the experiment according to method described by Trinder [34].

### 2.13. Statistical Analysis of the Blood Glucose Level

Results of the blood glucose level were expressed as mean ± standard error of the mean. The data were statistically analyzed using analysis of variance (ANOVA) with Duncan Multiple Range Test comparisons versus control groups. The values of *p* < 0.05 were considered as significant according to Duncan et al. [35].

## 3. Results and Discussion

### 3.1. The Physical Properties and Yields of *Senna occidentalis* Root

The results of physical properties such as consistency, color, and extractive value percentage yields of successively extracted in an order of nonpolar to polar solvent based on increasing degree of polarity are reported in Table 1. High values were found in polar solvent methanol (MeOH) followed by intermediate polarity ethyl acetate (EtAc) and nonpolar n-hexane (*n*-hex), respectively. Low *n*-hexane extractive value signifies the presences of amounts of fats, lipids, and some steroids in the plant materials and may indicate the addition of exhausted material, adulteration or incorrect processing during drying, or storage or formulating [1].

### 3.2. Thin-Layer Chromatography

**Figure 2:**
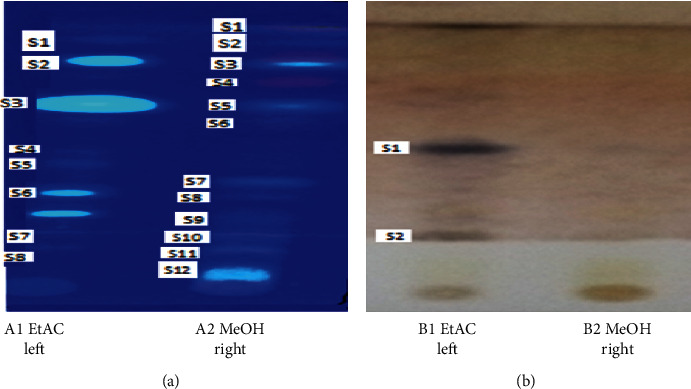
TLC separation of different extracts for chemical constituents of *Senna occidentalis* root by using solvent system toluene: ethyl acetate with the ratio 70 : 30. Keys: (a) before spray under UV and (b) after spray with anisaldehyde sulpharic acid reagent. S≡ spot of separation.

The results of the Thin-Layer Chromatography (TLC) separated the various phytochemical components in ethyl acetate and methanol extracts of *Senna occidentalis* root, by using solvent system toluene: ethyl acetate in the ratio (70 : 30) as described by Fried and Sherma [36] and Wagner and Bladt [37].  Figure 2, before spray: under UV (254 nm) light, several spots were seen with the *R*_f_ values colors at sample *A*1 Left representing the Et. Ac extract of root was given eight spots (S) with numbers from upper to the lower: *S*1 = 0.93 (pale blue fluorescent), *S*2 = 0.85 (green fluorescent), *S*3 = 0.67 (green fluorescent), *S*4 = 0.46 (sky blue fluorescent), *S*5 = 0.37 (green fluorescent), *S*6 = 0.28 (green fluorescent), *S*7 = 0.21 (light sky blue fluorescent), and *S*8 = 0.16 (light sky blue fluorescent) color. Sample A2 Right represents that the MeOH extract of root gave twelve spots with *R*_f_ values *S*1 = 0.98 (pale red fluorescent), *S*2 = 0.93 (pale blue), *S*3 = overlapping between 0.87 to 0.85 (Orange to green fluorescent), *S*4 = 0.75 (pale red fluorescent), *S*5 = 0.67 (blue bright sky fluorescent), *S*6 = 0.46 (blue fluorescent), *S*7 = 0.42 (light sky blue fluorescent), *S*8 = 0.38 (light sky blue fluorescent), *S*9 = 0.28 (light sky blue fluorescent), *S*10 = 0.21 (light green fluorescent), *S*11 = 0.16 (arc light green fluorescent), and *S*12 = 0.08 (arc green fluorescent). These colors indicated the presence of constituents may be with *R*_f_ values 0.38 (white sky blue to white green fluorescent), 0.44 (red), 0.88 (violet) alkaloids (white blue fluorescent), phenolic compounds (blue to white green fluorescent), negligible amount of alkaloids (very weak intensity), or saponins with glycosides in the roots with *R*_f_ (0.18, 0.32 (both orange) and a blue fluorescent spot *R*_f_ (.0.16, 0.32, 0.72, and 0.97) respectively, and these could not be isolated; but, on spraying with anisaldehyde sulpharic acid reagent and heating plate (B) for ten minutes at 110°C, spots of purple color appearing at grey dark violet colors were detected with two *R*_f_ values between 0.50 and 0.21 and constituents may be tannins or triterpine and phytosterol; Wagner and Bladt [37]. These colorless ones in the same plate constituents may be oils (Volatile oil and fixed oils) or protein and (dark brown) cardiac glycosides with the *R*_f_. (0.88, 0.60, 0.38, 0.32, 0.23, and 0.18. Generally MeOH and EtAc were observed to have a good manner of colored spots under UV light but showed less color toward the anisaldhyde sulphate reagent. Constituents may be stilbenes or pigments (0.96 with red or pink color). Constituents may be alkaloids with Rf 0.74 (no color) and 0.70 (no color). Constituents may be terpenoids and saponins with Rf 0.62 (dark blue), 0.61 (dark violet blue), 0.48 (dark blue), and 0.42 (dark blue violet). Flavonoids may be with Rf 0.32 (orange), 0.21 (orange), and 0.11 (dark blue orange). The chromatogram revealed a mixture of compounds which exhibited different colored reactions with *R*_f_ values: terpenoids (purple or blue violet), flavonoids (yellow, pinkish or orange), stilbenes (bright red to dark pink color), and proanthocyanidins (pink color). These results agreed with the chemical constituents reported by Wagner and Bladt [37] and Tsakala et al. [38].

### 3.3. Results of Phytochemical Screening of *Senna occidentalis* Root

**Figure 3:**
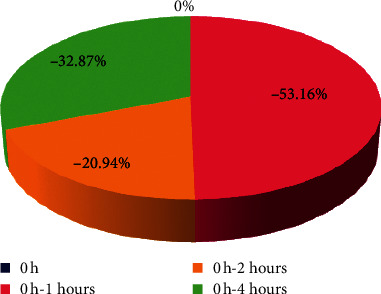
Effects of the ethanolic extract of *Senna occidentalis* (L.) roots part on glucose-loaded induced hyperglycemia in rats when compared within groups to fasting.

The general results of phytochemical screening are shown in Table 2. Tests supplementary Figure 1 showed the different testing of preliminary phytochemical screening of *Senna occidentalis* root; it gave the colors with few modifications according to the colors of extracts indicating the presence of the high concentrations of the various active phytoconstituents metabolites as follows: alkaloids (Dragendorff's Orange brown, Mayer's Yellow to creamshies, Hager's Yellow, and Wagner's reddish brown with precipitations), triterpenes (Liebermann–Burchard deep purple/violet and Salkowski deep red to brown color ring at the junction of the two layers), tannins (ferric chloride blackish blue to violet and salt gelatin white with precipitations), saponins (white foam at the upper layer persisting for 10 minutes), flavonoids (sodium hydroxide yellow, ammonium hydroxide deep yellow, Aluminum Chloride yellow-creamshies and magnesium turnate piece-sulfuric acid reddish), and sugars (Molisch's violets to green ring at the junction of the two layers; Benedict's few reducing monosaccharides; and Barfoed's high reducing disaccharides) followed by a moderate amount of proteins (Biuret violet and Coumarins blue fluorescence under UV light with KOH alcoholic), but low amount of amino acids (Ninhydrin purple color and xanthopreotic yellow-orange) and moderate amount of aromatic amino acids may be produced from degradation of proteins and glycosides (Anthraquinane pink-red and Cardiac A brown ring), respectively, in this part. Similar to the previous studies on other parts of this plant by Lawal et al. [39], on *S*. *occidentalis* leaves, the results found show that the leaves extracts contain glycosides (anthraquinones and cardiac), phenolic compounds such as terpene (has antivirial properties), flavonoids (have antiflammation and antioxidant), tannins (for wound healing), coumarins, and saponins with absence of alkaloids (have bloods pressure decrease and nervous system balancing). Also, similar findings were reported in [8] in the other species of the same family. According to TLC results in Figure 3(a), tthyl acetate and methanol extracts of this plant, under 254 nm UV light, showed the presence of eight and twelve spots, respectively, where different *R*_f_ values in colors may be flavanoids (orange to red yellow fluorescent), alkaloids (white blue fluorescent), and phenolic compounds (blue to white green fluorescent) and, after spraying with 1% anisaldhyde-sulphate reagent, showed few colored spots in the Et Ac extract and colorless spots in the MeOH extract with *R*_f_ values; Figure 3(b) suggests the presence of many compounds in the extracts. The Et Ac and MeOH extracts were subjected to the phytochemical screening for the detection of various plant constituents. It was found that terpenoids, flavanoids, and phenolic compounds were present as the major active principle [36, 37]. The active principles of many drugs found in plants are secondary metabolites, which are widely used in traditional medicine to treat various ailments. Galib et al. [24] mentioned that *Senna occidentalis* root could be used as larvicidal and antidiabetic.

### 3.4. Physiochemical Anaylsis

The nutrition composition percentage of plant powder of *S*. *occidentalis* root, Table 3, was determined with the high percentage of physiochemical which was found to be in dry matter (98%) followed by the nitrogen-free extract (69.6%), curd fiber (14.5%), curd proteins (8.15%), ether extract (3.75%), and both ash and moisture (2%). Many plant proteins are low in one of the essential amino acids, but a combination of plant proteins such as grains with pulse or seeds may give a high-quality protein which is just as good as the protein from animal foods.

### 3.5. The Results of the Minerals Composition

Table 4 showed the mineral composition *Senna occidentalis* root, and high concentrations values were found in potassium (43 mg/l or ppm) followed by phosphorous (28.5 mg/l), calcium (15 mg/l), sodium (3.65 mg/l), and magnesium (0.145 mg/l). Similar findings were reported by Agbugui et al. [40] in the other species of the same family. He showed these elements from a part of the rigid body structure, soft tissue, and body fluids of most vertebrates, and Anhwange et al. [41] reported that, in general, minerals work in combination with each other and other nutrients; therefore, deficiency of any mineral may cause health problems. Sodium and potassium maintain water balance in cells, and they are important for nerve impulse transmissions and stimulation of normal movement of the intestinal tract. Glew et al. [42] reported that magnesium is essential because it maintains, repairs cells, and provides energy and its deficiency may result in vertigo, convulsions, nervousness, and heat palpitation. It also assists the muscles to keep reservoir of oxygen and increases the body's resistance to infection. Its deficiency results in anemia, tiredness, insomnia, and palpitations.

### 3.6. Cytotoxicity Bioassays (Larvicidal Activity) of *Senna occidentalis* Root

The results of cytotoxicity bioassays with n-hexane, ethyl acetate, and methanol from the root extracts showed good larvicidal activities for all extracts; high mortality was observed with high concentration (1 g/L) representing 1000 ppm dose of methanol extracts, and low mortality was observed in low to moderate concentrations (0.01 and 0.1 g/L) representing 10 ppm and 100 ppm. 100% mortality was not obtained at all concentrations. In general, n-hexane and ethyl acetate were showed safe effects and methanol showed moderate effect; Table 5. Kumar et al. [43] showed the significant effect at other doses than that reported in this study, 100% mortality effect of petroleum ether and butanol of larvacidal activity of *C*. *occidentalis* against mosquito *Culex quinquefasciatus* at (0.2 and 0.3 g/L) representing 200 ppm and 300 ppm, respectively.

This studies of larvicidal activity of *Senna occidentalis* root n-hexane, ethyl acetate, and methanol extracts had LD_50_ values 1564721 ppm, 1412.54 ppm, and 1412.54 ppm, respectively, and were carried out against 3^rd^ instar of *Culex quinquefasciatus* mosquito larvae (Table 5), which revealed that the results of the lethal doses determined in both n-hexane and ethyl acetate extracts were larvicidal significant (*p* < 0.05) and highly effectively safe for mosquito larvae with increased LD_50_ values of 1564721 and 1412.54, respectively, and this indicates that nonpolar (*n*-hexane) and intermediate polar ethyl acetate have more potent activity to larvae. The methanol extract showed least effective larvicidal significant (*p* < 0.05) moderate effect for mosquito larvae with an LD_50_ value 257.54 ppm.

In Table 5, the aquatic immature larvae stage is recognized as the most vulnerable and best control strategy to effectively reduce mosquito population densities during infestations. *Senna occidentalis* root displayed good larvicidal effect considered to larvae safe (nontoxic) control by the solvent DMSO with an LD_50_ 726129 × 10^11^ at all concentrations according to the modification of Meyer et al. [44] and Duke et al. [45].

Yadav et al. [19] and Duke et al. [45] previously reported that the phytochemicals screening possess insecticidal activity which are present in plants of this genus, and it could be responsible to the larvicidal activity of *Senna occidentalis* root in n-hexane, ethyl acetate, and methanol extracts or it may be due to containing such phytochemicals and related compounds that display a role in plant defense against pests and insect and might have been responsible for larval deaths at high concentrations.

### 3.7. Effects of the Roots Supplement on the Blood Sugar Level (BSL)

**Figure 4:**
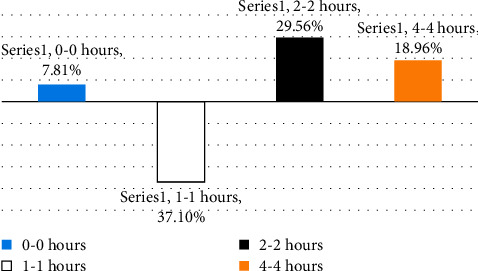
Effects of ethanolic extract of *Senna occidentalis* (L.) roots part on glucose-loaded induced hyperglycemia in rats when compared within groups to standard glibenclamide 10 mg/kg.

In the present study, the effects of the ethanolic extract of *Senna occidentalis* (L.) roots were determined on blood glucose levels of all animals treated with the supplement and were seen to reduce significantly (*p* < 0.05) by about 58.25%, 48.59%, and 41.93%, respectively, when compared with that of the untreated diabetic control group (Table 6) and cause a favorable hypoglycemic activity when compared to fasting glucose levels within groups (Figure 3) significant reduction by 53.15%, 20.94%, and 32.87%, respectively, but with standard glibenclamide 10 mg/kg significant reduction only between one hour with the value 37.10% and a significant increasing for the other 29.56% and 18.96%, respectively (Figure 4). This study proved the use of *Senna occidentalis* roots in traditional medicine for the treatment of diabetes and showed that *Senna occidentalis* roots supplement has good potent hypoglycemic effects which may be due to its high contents of active phytochemical constituents such as flavonoids that was reported in most medicinal plants with hypoglycemic and antidiabetic properties. This finding agrees with those of Gidado, et al. [4] and Verma et al. [8].

The phytochemical profiling of *Senna occidentalis* root extracts presented in this study revealed a diverse range of the bioactive phenolics compound. Some of the identified bioactive phenolics were reported by several authors for antidiabetic activities; Valle et al. [46], Can et al. [47], and Mohammed et al. [48]. Besides, the larvicidal activity was carried out against *Culex quinquefasciatus* mosquito larvae, which showed that the methanol extract showed moderate effects and n-hexane and ethyl acetate extracts showed safe effects. The larvicidal activities clearly showed safe effects in this part at high dose required for insecticidal action (1000 ppm), but the methanol extract is lower than doses used in animal study (200 ppm) for diabetic action.

This was an attempt to identify the potent antidiabetic and pesticides compounds from *Senna occidentalis* root. This plant is widely distributed, and it can be predicted that the potent antidiabetic and larvicidal activity of *Senna occidentalis* root is due to presence of compounds that have potential to be used in local communities for antidiabetic activity and also used as mosquito control during infestation seasons, thus promoting the use of natural antidiabetic and pesticides. It is concluded that *Senna occidentalis* (L.) in parts showed higher potent hypoglycemic activity in glucose-loaded diabetic rats than other plants. However, further effects can be investigated by the increase in the treating dose of the extract, and other pharmacological studies should be performed, specifically on dose 200 mg/kg, which was presented as the antidiabetic activity. Isolation and identification of more bioactive compounds were highly recommended. *In vivo* studies are needed to be conducted on these active ingredients to study the mechanism of action by which these compounds exert their effect as an antidiabetic activity. I recommended studying the antidiabetic activity using alloxan-inducing diabetic type 2 in same dose 200 mg/kg and histopathological in animal by the same manner. Gidado et al. [4] and Verma et al. [8] they reported in their previous studied on *Senna occidentalis* leaf part and whole parts showed *β*-cell regenerating potential as depicted by the histopathological studies of the pancreatic tissue, even though some cytotoxic agents such as the cytotoxic saponins may be present in the supplement which is capable of causing damage to both the pancreas and liver and induced hepatotoxicity.

## Figures and Tables

**Table 1:** The physical properties of *Senna occidentalis* root.

Solvents of extraction	Consistency	Color	Yields% ± SD
*n*. Hexane	Semisolid	Yellow	0.046% ± 0.00
Ethyl acetate	Semisolid	Brown	0.15% ± 0.00
Methanol	Solid powder	Dark brown	1.13% ± 0.00

**Table 2:** Results of phytochemical screening of *Senna occidentalis* root.

Metabolites	Tests	Methanol

Alkaloids	Dragendorff's	++++
	Mayer's	++++
	Hager's	++++
	Wagner's	++++

Coumarins	KOH/U. V.	++

Flavonoids	NaOH/KOH	+++
	NH3OH con.	+++
	ALCL3 (flavones or and chalcone).	+++
	Mg/HCL (flavones/flavanol glycosides)	−

Glycosides	Anthraquinane	+
	Cardiac	+

Saponins	Foam	+++

Sterols/triterpene	Liebermann–Burchard	−/++++
	Salkowski	−/++++

Tannins	Ferric chloride	+++
	Salts gelatin	++

Sugar	Molisch's	+++
	Fehling's/Benedict's	+
	Barfoed's	+++

Amino acids	Ninhydrin	+
	Xanthopreotic	++

Proteins	Biuret	++

Lipids	Sudan III	–

++++ = very high amount, +++ ≡ high amount, ++ ≡ moderate amount, + ≡ low amount, ± ≡ trace amount or absent, and − = absent.

**Table 3:** Physiochemical (proximate analysis) of *Senna occidentalis* root.

Proximate analysis	Values (% ±SD)	Proximate analysis	Values (% ±SD)
Dry matter	98 ± 0.00	Crude fibers	14.5 ± 0.71
Moisture	2 ± 0.00	Nitrogen-free extracts	69.6 ± 1.62
Ash	2 ± 0.00	Reducing sugar	6.5 ± 0.71
Ether extracts	3.75 ± 0.07	Total sugar	26 ± 1.41
Crude protein	8.15 ± 0.01	Total tannin	25 ± 2.83

**Table 4:** Mineral composition values of crude powder of *Senna occidentalis* roots.

Potassium (mg/l ±SD)	Phosphorus (mg/l ±SD)	Calcium (mg/l ±SD)	Sodium (mg/l ±SD)	Magnesium (mg/l ±SD)
43 ± 0.01	28.5 ± 0.00	15 ± 0.01	3.65 ± 0.00	0.145 ± 7.07

Keys: = means of duplicates values.

**Table 5:** The effects of different concentrations of n-hexane, ethyl acetate, and methanol extracts on *Culex quinquefasciatus* mosquito larvae mortality rate.

Extract	Con. (ppm)	Total	^*∗*^Death	^*∗*^Survive	LD50	Remark
*n*-Hexane	10	30	1	29	1564721	Safe
	100	30	3	27		
	1000	30	4	26		

Ethyl acetate	10	30	3	27	1412.50	Safe
	100	30	3	27		
	1000	30	14	16		

Methanol	10	30	2	28	257.24	Moderate
	100	30	4	26		
	1000	30	27	3		

Keys: ^*∗*^≡ means of triplicates values; conc. ≡ concentrations; (ppm) ≡ part per million (mg/l).

**Table 6:** Effects of *Senna occidentalis* (L.) Roots of 80% ethanolic extracts on glucose-loaded rats.

Treatments	Bloods glucose levels mg/dl mean ± S. D.			
	Time			
Groups and dose	0 h	1 hours	2 hours	4 hours
Group I “normal vehicle 2% water”	68.33^*∗*^^b^ ± 8.50	63.67^*∗*^^c^ ± 2.52	69.33^*∗*^^c^ ± 11.68	57.67^*∗*^^c^ ± 3.21
Group II glucose loading 2 g/kg body wt “positive control”	89.00^a^ ± 11.27	114.00^a^ ± 13.11	138.33^a^ ± 13.32	132.67^a^ ± 30.75
Group III glucose loading 2 g/kg + standard glibenclamide 10 mg/kg.	93.67^a^ ± 11.6 (5.25%)↑	75.67^*∗*^^b,c^ ± 14.15 (−33.62%)↓	62.00^*∗*^^b,c^ ± 7.81 (−55.18%)↓	57.33^*∗*^^b,c^ ± 3.79 (−56.79%)↓
Group VI glucose loading 2 g/kg + EtOH of *S*. *o* roots 200 mg/kg.	101.6^a^ ± 18.4 (14.15%)↑	47.6^*∗*^^b,c^ ± 15.6 (−58.25%)↓	80.33^*∗*^^c^ ± 4.5 (−41.93%)↓	68.2^*∗*^^b,c^ ± 6.9 (−48.59%)↓

*n* = 6, ^*∗*^*a* = <0.05 indicates a significant difference compared with diabetic control; ^*∗*^*b* = (*p* < 0.05) indicates a significant difference compared with diabetic control; ^*∗*^*c* = (*p* < 0.05) indicates a significant difference compared with standard drug (glibenclamide), % = level_2_-level_1_/level_1_X100 compared with diabetic control. *S*. *o* *=* *Senna occidentalis*.

## Data Availability

We have already included all data in the manuscript, the lab and data, phytochemical screening, cytotoxicity-larvicidal activity, antidiabetic activity, and nutrients and minerals.

## Supplementary Materials

Supplementary Materials Supplementary Figure 1 showed different testing of preliminary phytochemical screening of *Senna Occidentalis* root including acids (2% HCL) alkaloids, tannins screening, triterpenes and sterols, and saponins screenings.
