# Supplementary material for: Physiochemical, Insecticidal, and Antidiabetic Activities of Senna occidentalis Linn Root
Source: Biochem Res Int. 2020 Sep 15;2020:8810744. doi: 10.1155/2020/8810744 (PMC7512105; doi:10.1155/2020/8810744)
Supplement: Supplementary Materials — Supplementary Figure 1 showed different testing of preliminary phytochemical screening of Senna Occidentalis root including acids (2% HCL) alkaloids, tannins screening, triterpenes and sterols, and saponins screenings. [file 8810744.f1.doc]

**Different testing of Preliminary Phytochemical screening of *Senna Occidentalis* Root**


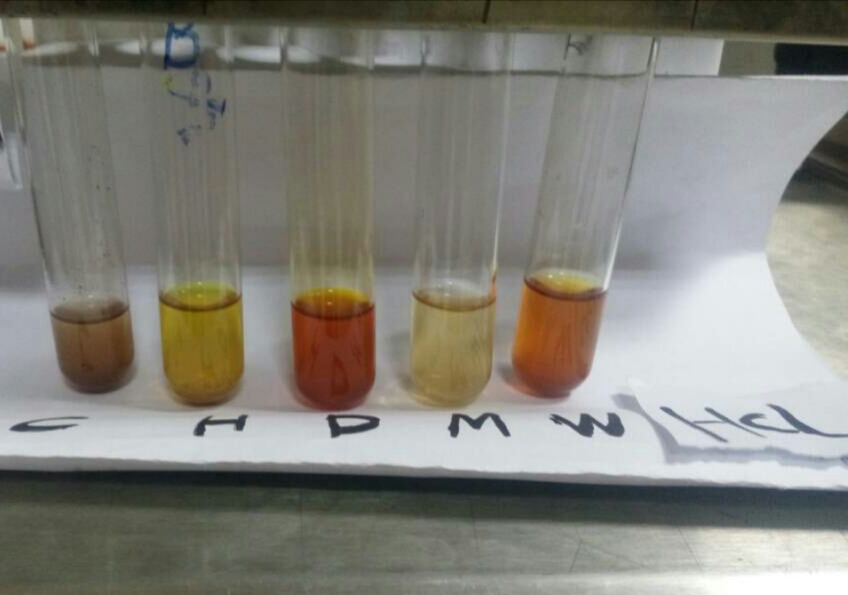


Control Hager’s Dragendorff’s Mayer’s Wagner’s

**Acidic (2%HCL) Alkaloids screening of *S. occidentalis* Root**


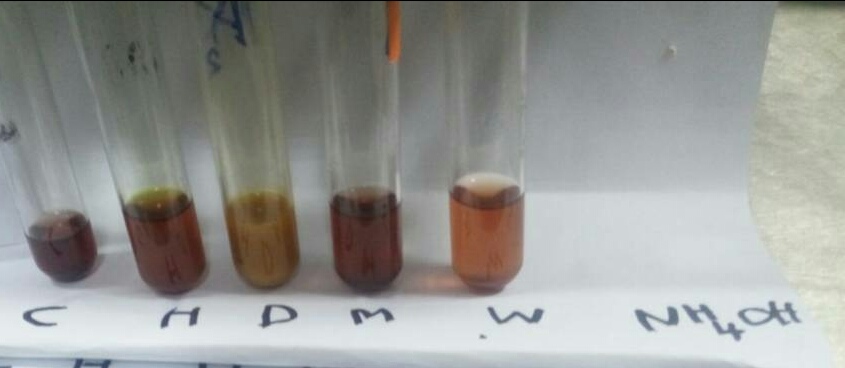


Control Hager’s Dragendorff’s Mayer’s Wagner’s

**Basic Alkaloids** (**10% NH_4_OH) screening of *S. occidentalis* Root**

**
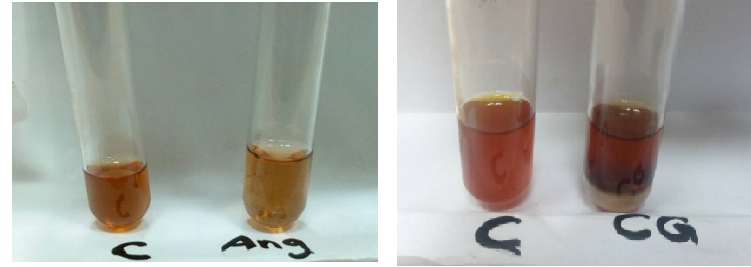
**

**Anthraquinane (A) Cardiac (B)**

**Glycosides screening of *S. occidentalis* Root**


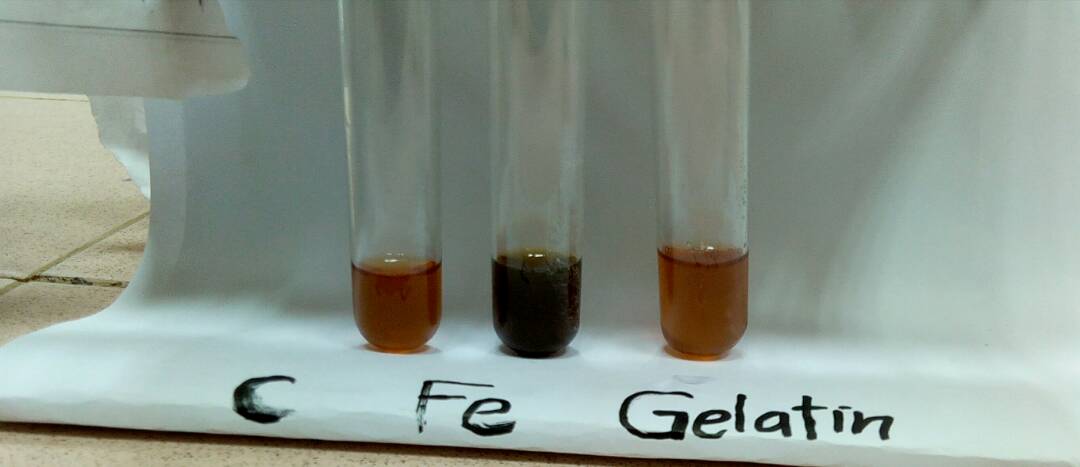


Control FeCl_3_ Gelatin

**Tannins screening of *S. occidentalis* roots**


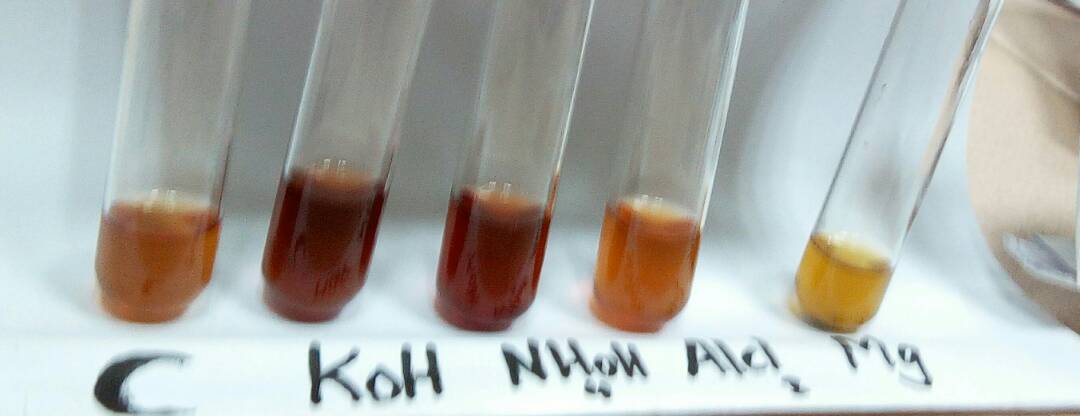


**Flavonoids screening of *S. Occidentalis* roots**


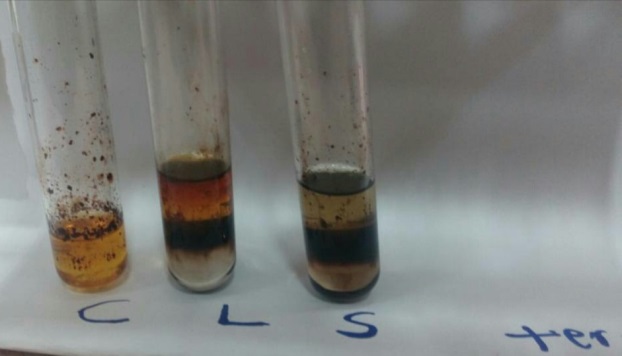


Control Liebermann Salkowski

**Triterpenes and sterols screening of *S. occidentalis* roots**


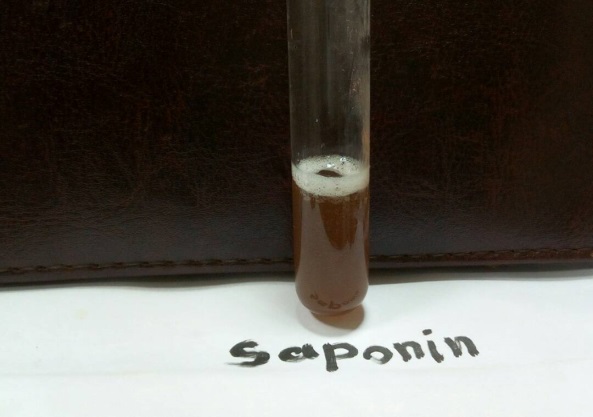


**Saponins screening of *S. occidentalis* roots**
